# Effect of In Vitro Micropropagation on the Chemical, Antioxidant, and Biological Characteristics of *Senecio nutans* Sch. Bip., an Endemic Plant of the Atacama Desert Andean Region

**DOI:** 10.3390/plants13060755

**Published:** 2024-03-07

**Authors:** Claudio Parra, Patricio Muñoz-Torres, Hugo Escobar, Mario J. Simirgiotis, Gabriela Contreras-Contreras, Álvaro Ruiz-Fernández, Cristian Maulen, Maximiliano Martínez-Cifuentes, María Salomé Mariotti-Celis

**Affiliations:** 1 Departamento de Química Orgánica, Facultad de Ciencias Químicas, Universidad de Concepción, Concepción 4070371, Chile; cmaulen2018@udec.cl; 2 Facultad de Ciencias Agronómicas, Universidad de Tarapacá, Arica 1000000, Chile; pmunozt@academicos.uta.cl (P.M.-T.); hescobar@academicos.uta.cl (H.E.); 3 Facultad de Ciencias, Instituto de Farmacia, Universidad Austral de Chile, Valdivia 5110566, Chile; mario.simirgiotis@uach.cl; 4 Escuela de Nutrición y Dietética, Facultad de Medicina, Universidad Finis Terrae, Santiago 7501015, Chile; gcontreras@uft.cl; 5 Computational Biology Lab, Fundación Ciencia & Vida, Santiago 8580702, Chile; aruiz@dlab.cl

**Keywords:** acetophenone, antibacterial, BDE, chachacoma, UHPLC-DAD

## Abstract

The species *Senecio nutans* Sch. Bip., commonly called “chachacoma”, is widely used as a medicinal plant by the Andean communities of Northern Chile. Ethanolic extracts of *S. nutans* and the main compound, 4-hydroxy-3-(3-methyl-2-butenyl) acetophenone, have shown interesting biological activity. However, due to the high-altitude areas where this species is found, access to *S. nutans* is very limited. Due to the latter, in this work, we carried out micropropagation in vitro and ex vitro adaptation techniques as an alternative for the massive multiplication, conservation, and in vitro production of high-value metabolites from this plant. The micropropagation and ex vitro adaptation techniques were successfully employed, and UHPLC-DAD analysis revealed no significant changes in the phenolic profile, with acetophenone **4** being the most abundant metabolite, whose antioxidant and antibacterial activity was studied. Independently of the applied culture condition, the ethanolic extracts of *S. nutans* presented high activity against both Gram-positive and Gram-negative bacteria, demonstrating their antimicrobial capacity. This successful initiation of in vitro and ex vitro cultures provides a biotechnological approach for the conservation of *S. nutans* and ensures a reliable and consistent source of acetophenone **4** as a potential raw material for pharmacological applications.

## 1. Introduction

**Figure 1:**
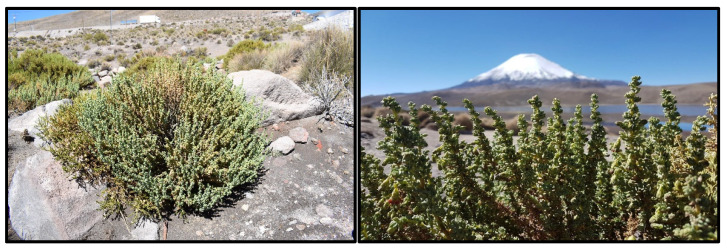
*Senecio nutans* collected in the Atacama Desert Andean Region at 4566 m.a.s.l.

The Puna ecoregion extends from South-Central Peru to Northern Argentina and Chile, with an altitude ranging from 3000 to 5000 m above sea level. In this habitat, it is possible to find natural products with therapeutic potential, such as *Senecio nutans* Sch. Bip. (synonyms: *Senecio graveolens* Wedd., *Senecio graveolens* var. *Psiloachaenius* Cabrera), from the family Compositae (Figure 1). *Senecio nutans* is known in Chile by the vernacular name “chachacoma”. This plant is a perennial shrub about 20–60 cm high, and local Andean communities widely use it for altitude or mountain sickness, characterized by headaches, dizziness, vomiting, and fatigue [1,2,3,4,5]. Due to the high-altitude areas where this species is found, access to *S. nutans* is very limited. Recently, and due to these geographical characteristics, a detailed study on its chemical composition has been reported [6].

Some investigations have shown that ethanolic extracts of *S. nutans* have antimicrobial and antibacterial activity [4,7,8,9]. These notable properties are associated with the different types of secondary metabolites present in the plant. Mainly, the *Senecio* species are rich in antioxidant compounds such as polyphenols (phenylpropanoids, 3,5-di-O-caffeoyl quinic acid, 3,4-di-O-caffeoyl quinic acid, chlorogenic acid methyl ester, caffeic acid and chlorogenic acid, tannins, and flavonoids) [7,10], terpenes [4], and *p*-hydroxyacetophenone derivates [11,12,13]. *p*-Hydroxyacetophenone derivates are the most abundant compounds in the aerial parts of *S. nutans* species [3,12,13]. Other authors found several types of *p*-hydroxyacetophenones in *S. nutans*, such as 4-hydroxy-3-(isopenten-2-yl) acetophenone, 4-hydroxy-3(3′-hydroxyisopentyl) acetophenone, and 4-hydroxy-3-(3-methyl-2-butenyl) acetophenone (AC) [13]. Studies with Gram-negative bacteria have demonstrated that 4-hydroxy-3-(3-methyl-2-butenyl) acetophenone and acetophenone derivates are ideal compounds to apply in biological assays [9]. Specifically, AC has demonstrated antimicrobial activity [9] and antibacterial activity [14]. Synthetic derivates of AC can be obtained via carbonyl reactions, which expands the potential for the investigation of the influence of this type of derivate in biological studies [12].

Most of the *S. nutans* plant material consumed by local inhabitants is collected from the wild, leading to the overexploitation and predation of this plant. The future of this plant in a global climate change scenario, such as the one that is occurring today, foresees this and other species’ disappearance [15]. Decades ago, the distribution of chachacoma was much more extensive, and no risk to its survival prevailed [1,16]. The lack of knowledge about the cultivation of this species, its overexploitation and predation, and the effects of climate change have led countries like Peru to protect this species from vulnerability [17]. In this context, techniques based on plant tissue culture have emerged as an alternative for the massive multiplication, conservation, and in vitro production of high-value plant species’ secondary metabolites worldwide [18,19]. Micropropagation in vitro as a tissue culture remains the best strategy for the production of complex natural products, especially if the plant source material is overexploited, slow-growing, or low-yielding [20].

Based on the above, this study evaluated the impact of in vitro micropropagation on the content of secondary metabolites (polyphenols and acetophenone derivates), the antioxidant properties, and the antibacterial activity of ethanolic extracts obtained from ex-vitro-acclimated *S. nutans* plants and their majority compound. The primary objective was to develop a protocol that enables the scaled production of this native plant, thereby preventing its extinction while preserving its bio-activity.

## 2. Results and Discussion 

### 2.1. In Vitro Cultivation and Ex Vitro Acclimation of S. nutans

The Atacama Desert Andean Region stands out for the significant occurrence of exotic endemic species at risk of extinction, which are potential sources of nutraceuticals and functional ingredients [21,22,23]. In this sense, to conserve these rare and endangered plants, micropropagation or in vitro cultivation offers a biotechnological approach to control growth independent of seasonal and climatic variations. 

**Figure 2:**
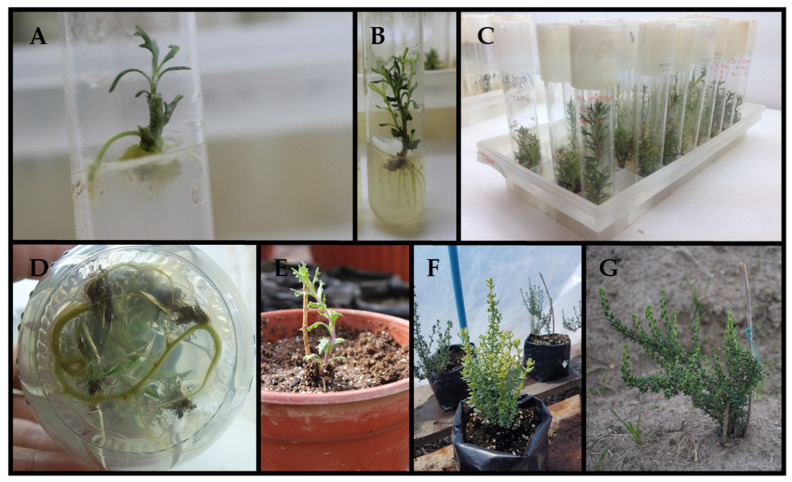
In vitro propagation and ex situ conservation of *Senecio nutans*. (**A**) Shoot induction in nodal explant in MS medium supplemented; (**B**,**C**) shoot proliferation in MS medium supplemented; (**D**) In vitro rooting in MS medium; (**E**) Potted plants inside culture room for gradual acclimatization; (**F**) Well acclimated plants showing promising growth after acclimatization; (**G**) Well acclimated plants in the field.

This study focused on micropropagation by stem cuttings due to the unsuccessful seed germination of *S. nutans* (Figure 2A). The regenerated microplants in vitro developed strong roots and abundant leaf biomass, featuring shoots of 1.5 cm in length and a well-developed root system (Figure 2C,D). The initial stage of in vitro microplate acclimation in a controlled environmental phytotron chamber was successful. The survival rate of acclimated ex vitro *S. nutans* plants was relatively high, reaching 85% (Figure 2E). The percentage of surviving plants remained consistent throughout the process, including greenhouse adaptation (Figure 2F) and transplantation into the wild soil in the experimental field, in the same area where wild samples were collected (Figure 2G). With plants acclimated ex vitro, the impact on the content of secondary metabolites of the plant material was evaluated in two stages of its development (in vitro and ex vitro) concerning wild samples of *Senecio nutans*.

### 2.2. Phenolic Profile of In Vitro, Ex Vitro, and Wild S. nutans

**Figure 3:**
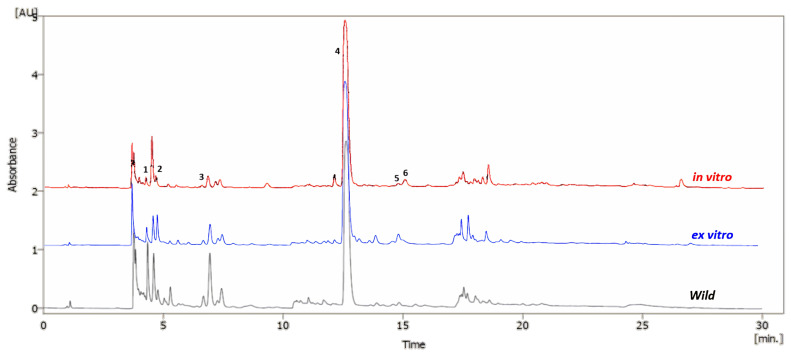
UHPLC-DAD chromatogram of *Senecio nutans* ethanolic extract at 280 nm in wild, in-vitro-cultivated, and ex-vitro-adapted plants.

**Figure 4:**
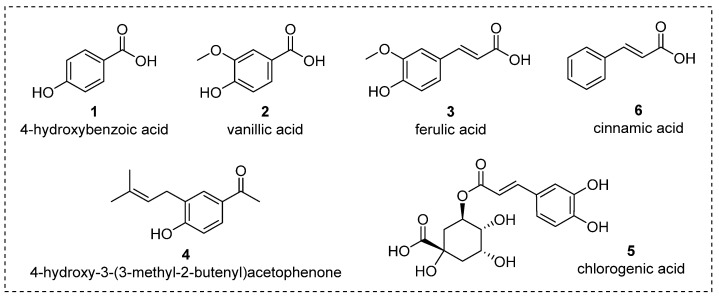
Chemical structures of the main secondary metabolites identified in the extracts.

To elucidate the metabolic changes occurring in *S. nutans* plants throughout the in vitro and ex vitro preservation processes, we employed UHPLC-DAD analysis. We successfully identified six distinct compounds, comprising five phenolic compounds and one acetophenone. Remarkably, there were no notable qualitative distinctions in the metabolic profiles between in-vitro-cultivated and ex-vitro-acclimatized plants, except for two unidentified compounds, which were exclusively detected in the chromatograms of ex-vitro-acclimated plants (Figure 3 and Table 1). Recently, the chemical characterization of the hydroalcoholic extract of *S. nutans* detected 51 metabolites [6]. However, this study detected and quantified, for the first time, the presence of 4-hydroxybenzoic acid, vanillic acid, and cinnamic acid in the ethanolic extracts obtained from ex vitro and wild *S. nutans* (Figure 4).

AC (compound **4** in Figure 4) emerged as the predominant secondary metabolite in all sample extracts. AC has been recognized for its diverse biological properties, encompassing cytotoxic [24], antimicrobial [9], and cardiac contractility effects [3,6]. This compound has served as a starting material for the synthesis of numerous compounds with pharmacological potential, aimed at the preventative treatment of various diseases [12,25]. Considering that certain pharmaceutical and nutraceutical products often rely on plant-derived metabolites [20], these findings offer promise in ensuring a reliable and consistent source of AC as a potential raw material for such applications.

Table 1 shows the concentrations found for the different identified metabolites. Cinnamic acid, vanillic acid, and 4-hydroxybenzoic acid exhibit various biological properties, including antioxidant, antimicrobial, and antifungal attributes [26]. Additionally, cinnamic acid offers anti-inflammatory and blood-sugar-regulating effects, making it a valuable candidate for conditions like diabetes [27,28]. In contrast, vanillic acid can lower hyperglycemia, while 4-hydroxybenzoic acid and cinnamic acid are noteworthy for their neuroprotective potential [29]. The discovery of these novel metabolites in the ex vitro samples offers a promising foundation for the refinement of the protocol to enhance these compounds’ production. The controlled laboratory conditions and the initial work conducted at sea level might have contributed to the plant’s heightened expression of these metabolites. Notably, 4-hydroxybenzoic acid is among the newfound metabolites in the ex-vitro-adapted *S. nutans* sample chromatogram.

### 2.3. Phytochemical and Antioxidant Activity

#### 2.3.1. Comparative Determination of Total Phenols and Flavonoids

**Figure 5:**
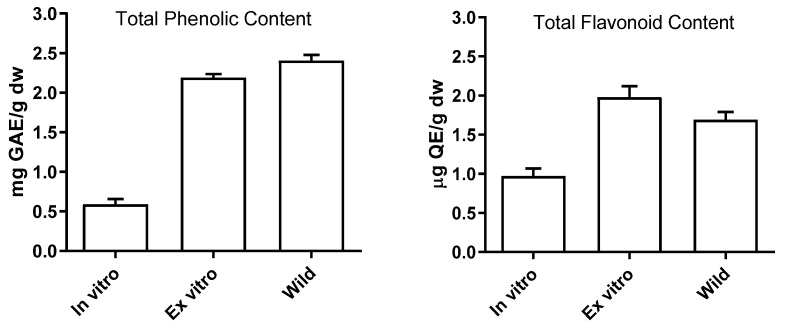
Total phenolic and flavonoid content in in-vitro-cultivated, ex-vitro-adapted, and wild *S. nutans* plants. GAE = gallic acid equivalents; QE = quercetin; dw = dry weight.

Phenolic compounds, known for their strong antioxidant properties, are secondary metabolites found in various plant species. These compounds are primarily synthesized in response to unfavorable environmental factors, both biological and non-biological, and their levels can vary significantly depending on the growing conditions. Analyzing the total phenolic and flavonoid content in *S. nutans* plants grown in different environments (in vitro, ex vitro, and wild) allowed us to observe the quantitative changes in metabolic content within plants of the same genotype. The highest phenolic content was found in the wild plants, followed by the ex-vitro-adapted *S. nutans* (2.41 ± 0.24 mg GAE/g DW and 2.19 ± 0.41 mg GAE/g DW, respectively; Figure 5). 

The flavonoid content exhibited a reverse pattern, with the highest levels found in ex-vitro-acclimated plants, followed by wild-grown plants (1.98 ± 0.33 µg QE/g DW and 1.69 ± 0.54 µg QE/g DW, respectively). In contrast, a significant, nearly fourfold decrease in total phenol content and a twofold decrease in flavonoid content were observed in in-vitro-cultivated *S. nutans* compared to the wild-growing genotypes. This decline in phenolic compounds was likely due to the aseptic culture conditions and mixotrophic nutrition provided to the micropropagated *S. nutans* plants in a controlled environment. Other researchers have also reported differences in the phytochemical content between wild and in-vitro-propagated plants [19]. These variations can be attributed to specific endogenous and metabolic physiological changes that occur in plants during the propagation process [30].

#### 2.3.2. Antioxidant and Radical Scavenging Activity of *S. nutans*

**Figure 6:**
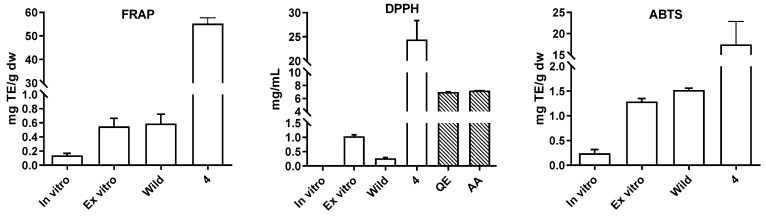
Antioxidant and radical scavenging potential of in-vitro-cultivated, ex-vitro-adapted, and in situ *S. nutans* plants and acetophenone. TE = Trolox equivalents; dw = dry weight.

Phenolic compounds have been acknowledged for their essential role as antioxidants, thanks to their unique structural characteristics and chemical behavior. Their ability to donate hydrogen allows them to scavenge free radicals and protect against reactive oxygen species (ROS) [31]. A positive correlation was observed between the phenolic content and ferric-reducing antioxidant power (FRAP), with the highest FRAP found in wild and ex-vitro-acclimated plants (0.59 ± 0.07 and 0.55 ± 0.02 mg TE/g DW, respectively). In-vitro-cultivated *S. nutans* plants showed approximately four times lower FRAP (0.14 ± 0.04 mg TE/g DW; Figure 6). Comparing the three extracts, the ex vitro plant extract exhibited significantly lower DPPH radical scavenging activity (1.04 ± 0.32 mg/mL) compared to the wild plant extract (0.27 ± 0.10 mg/mL). However, both *S. nutans* samples showed a lower DPPH uptake capacity than the quercetin (QE; 6.99 ± 0.41 mg/mL) and ascorbic acid (AA; 7.20 ± 0.97 mg/mL) standards in the DPPH assay. Notably, the in vitro sample did not display any DPPH radical scavenging activity. These results show the significant antioxidant potential of this plant; however, today, it is challenging to compare plant extracts or pure compounds’ antioxidant capacities, estimated by IC_50_ values, from different studies due to the variability in the DPPH concentration used in each methodology [32].

The highest ABTS radical scavenging activity was observed in the in situ wild and ex-vitro-adapted plants, with values of 1.52 ± 0.12 and 1.29 ± 0.23 mg TE/g DW, respectively. In contrast, the in-vitro-cultivated *S. nutans* exhibited a significantly lower capacity, approximately five times lower, with a value of 0.24 ± 0.02 mg TE/g DW (Figure 5. The antioxidant capacity of this species is significantly higher than that of other wild species growing under similar conditions in the arid Andean Region of Chile [33,34]. The observed higher antioxidant activity in ex vitro plants compared to in-vitro-grown plants can be attributed to the different stress levels experienced in these growth conditions [19,35]. The wild sample was more exposed to physical, climatic, and biological stresses, while the in vitro cultures were less vulnerable as they were kept under controlled conditions with nutrient media. These stress conditions stimulated the development of ROS, prompting plants to establish a robust antioxidant system for survival [35,36].

**Figure 7:**
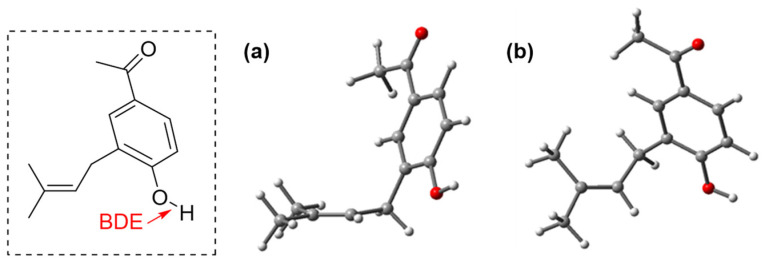
Optimization of the structure of the AC at the M06-2x/6-311+G(d,p) level. (**a**) View from the top of the double bond. (**b**) View from the side of the double bond.

On the other hand, we determined, for the first time, the antioxidant capacity of AC, since it is the main compound of the ethanolic extract of *S. nutans*, to demonstrate the value of this metabolite and its potential for large-scale production. The results for FRAP, ABTS, and DPPH (55.20 ± 2.30 mg TE/g DW, 17.44 ± 4.94 mg TE/g DW, and 24.44 ± 3.59 mg/mL, respectively) are shown in Figure 6. These results are promising, and, in this context, we conducted a computational analysis of the antioxidant capacity of this compound. The optimization of the structure of the compound was carried out at the M06-2x/6-311+G(d,p) level. Figure 7 presents the optimized structure of AC, which presents two coplanar parts united by a CH_2_ with an angle of 111.1° (the Cartesian coordinates of the compound are included in the Supplementary Materials). 

The antioxidant properties of a molecule can be evaluated by analyzing its ability to scavenge invading radicals. The nature of radical scavenging can be assessed using thermodynamic parameters, where the bond dissociation enthalpy (BDE) and ionization potential (IP) are the main ones. BDE is associated with the hydrogen atom transfer (HAT) mechanism, while IP is associated with the single electron transfer (SET) mechanism [37]. A decrease in BDE favors the HAT mechanism, while a decrease in IE favors the SET mechanism. Table 2 presents the calculated BDE for O-H bonds and IP for AC. 

These results show that AC tends to react earlier through the HAT mechanism over the SET mechanism. The latter suggests that the compound will be an effective radical-trapping antioxidant [38].

### 2.4. Antibacterial Activity

Table 3 presents the antimicrobial activity of extracts from in-vitro-micropropagated, ex-vitro-adapted, and wild plants of *S. nutans* and the major AC. These extracts demonstrated activity against both Gram-positive and Gram-negative bacteria. The minimal inhibitory concentration (MIC) tests showed activity ranging from 0.313 to 1.25 mg/mL of the extract. The minimum bactericidal concentration (MBC) ranged from 2.50 to 5.00 mg/mL of the extract, except for *Bacillus subtilis*, for which the MBC could not be determined due to its spore-producing nature. *Salmonella enterica* and *Staphylococcus aureus* were the most susceptible bacteria, requiring 0.313 mg/mL of the extract. No significant differences were observed in the antibacterial activity of the three extracts studied, except for slight variations in their effects on *E. coli* and *S. aureus*. Similar findings were reported by Salvador et al. [39], who observed lower antibacterial and antifungal activity in *Alternanthera maritima* extracts obtained from callus cultures compared to the adult plant. Moreover, the MIC test of isolated acetophenone showed activity ranging from 0.16 to 1.25 mg/mL of the compound. Meanwhile, the MBC ranged from 0.313 to 5.00 mg/mL of AC.

Our findings demonstrate that the extracts from both wild, ex-vitro-adapted (EV), and in vitro (IV) samples of *S. nutans* exhibited antibacterial activity against both Gram-positive and Gram-negative bacteria. These results differ from those reported by Santander et al. [9], who observed reduced activity against Gram-negative bacteria. Santander attributed the antimicrobial activity to the presence of AC, which may act by permeabilizing bacterial plasmatic membranes and inhibiting cell division through the suppression of teichoic acid synthesis [9]. The presence of various compounds, including acetophenone, in the *S. nutans* extract may contribute to its antimicrobial activity against both Gram-positive and Gram-negative bacteria.

## 3. Materials and Methods

### 3.1. Plant Material 

Nodal explants were taken from wild plants as starting material for the in vitro propagation of *Senecio nutans* Sch. Bip., which was collected in Chungara Lake, Parinacota Province (18°15′4.67″ S; 69°36′7.44″ W, 4566 m.a.s.l.), Northern Chile in October 2016 and was identified and deposited with the voucher specimen number UdeC-184934. 

### 3.2. In Vitro Micropropagation

Apical segments from the branches (4 cm long) of field plants were washed in water with detergent and then immersed in an antioxidant solution of 100 mg/L of citric acid and 300 mg/L of ascorbic acid for 20 min, followed by 1 min in 70% alcohol and 2.5% sodium hypochlorite with two drops of Tween 20 per 100 mL of disinfectant solution for 10 min, and then rinsed three times with sterile distilled water [40]. Three nodal explants were excised from the apical segments of branches of adult wild plants and cultured in 10 mL of modified MS [41] culture medium in 20 mm × 15 cm test tubes. MS medium was supplemented with different concentrations of 6-γ,γ-dimethylallylaminopurine (2-iP; 2.5 µM, 3.5 µM, 5.0 µM, 7.0 µM, and 10 µM) for the shoot induction and proliferation stage. The regenerated shoots (3 to 4 cm long) from actively growing cultures were excised and subjected to a rooting procedure on solid half-strength MS medium, supplemented with different concentrations of indole-3-butyric acid (IBA; 2.5 µM, 3.5 µM, 5.0 µM, 7.0 µM, and 10 µM). Both media also contained 3% sucrose, 100 mg/L myo-inositol, 0.5 mg/L thiamine-HCl, 0.5 mg/L pyridoxine, 0.5 mg/L folic acid, and 0.05 mg/L biotin. The MS medium was solidified with 0.6% (*w*/*v*) agar, and the pH of the medium was adjusted to 5.6 with 0.1 N NaOH and 0.1 N HCl before autoclaving at 115 °C for 15 min. All in vitro cultures were maintained in a growth room at 27 ±  2 °C under a 16/8 h light/dark photoperiod with 29 µE/m s illumination provided by cool white fluorescent tubes (Philips 40 W).

#### 3.2.1. Stages of Micropropagation

In this research, three experimental stages of the in vitro process were considered: (1) the establishment stage, which considers the establishment of explants in the culture media, which were obtained from adult wild plants, with the aim of inducing meristem sprouting; (2) the proliferation stage, to increase the number of shoots obtained in the first stage; (3) the rooting stage, which is aimed at inducing adventitious roots in the shoots obtained in the previous stages. The experimental design for each stage (establishment, proliferation, and rooting) was completely randomized, with six treatments of four repetitions of six explants each. The data were analyzed by ANOVA and Fisher’s multiple range tests (*p* ≤ 0.05). The variables, evaluated as percentages, were previously transformed to Bliss degrees.

#### 3.2.2. Acclimatization

All plants obtained from these experiments were removed from the test tubes, washed with tap water to remove the culture medium from the roots, and set in speedlings containing a peat moss substrate. Subsequently, they were transplanted into 1000 mL plastic containers containing a mixture of substrate with sand and peat moss (1:1), wet, at field capacity.

### 3.3. Phytochemical Analysis

#### 3.3.1. Extraction Preparation

Approximately 25 g of dried *S. nutans* aerial parts was pulverized and then extracted with 350 mL of 80% *v*/*v* ethanol/water in an ultrasonic bath for 15 min in the dark (70 mL, five times). The supernatant was concentrated in vacuo, and a brown extract (230 mg) was obtained. For HPLC analysis, 5 mg of the *S. nutans* extract was dissolved in 2 mL of HPLC-grade methanol, filtered (0.45 μm PTFE filter), and 10 µL injected into the instrument. For the phytochemical tests, 1 mg/mL was used, and 20 mg/mL of the stock solution was used for the antibacterial test.

#### 3.3.2. Determination of Total Phenol and Flavonoid Content

The total phenol content (TPC) was determined using the Folin–Ciocalteu method [42]. TPC quantification was performed based on the gallic acid standard curve and the results were expressed in mg gallic acid equivalents (GAE)/g dry weight. The total flavonoid content (TFC) was determined by the aluminum chloride colorimetric method [33]. TFC quantification was performed based on the quercetin standard curve and the results were expressed in mg quercetin equivalents (QE)/g dry weight.

### 3.4. Antioxidant Activity Assays

#### 3.4.1. Ferric-Reducing Antioxidant Power (FRAP)

The ferric-reducing antioxidant power of the samples was determined according to Parra et al. [42]. Data were expressed as µmol trolox equivalents (TE)/100 g DW. Trolox was used as a standard and ethanol 80% *v*/*v* as a blank.

#### 3.4.2. Free Radical Scavenging (DPPH)

The radical DPPH methodology was used, with some modifications [33]. An aliquot (150 µL) of different concentrations of extract (1.56–100 µg/mL) was mixed with 50 µL of 1 mM DPPH solution and incubated for 30 min at 25 °C. The absorbance was fixed at 517 nm, and the sample was shaken for 5 min and incubated for 30 min at 36 °C. The percentage of radical inhibition of DPPH was calculated according to Equation (1):

(1) $$\%=\frac{\left({Abs}_{\mathrm{control}}-{Abs}_{\mathrm{sample}}\right)}{{Abs}_{\mathrm{control}}}\times 100$$

Subsequently, a curve of the % inhibitory activity of DPPH versus the extract concentration was drawn, and the IC_50_ value was calculated.

#### 3.4.3. ABTS Method

The ABTS assay was performed by bleaching the cationic radical ABTS•^+^ as described by Echiburu-Chau et al. [33]. Results were expressed as micromoles of Trolox equivalents per 100 g dry sample (µmol TE/100 g DW).

#### 3.4.4. Computational Calculations

In the present work, density functional theory (DFT) calculations were carried out at the M06-2X/6-311+G(d,p) level, using the Gaussian 09 software (Revision a.01; Gaussian, Inc.: Wallingford, CT, USA) [43]. No imaginary vibrational frequencies were found at the optimized geometries, indicating that they were the true minimal of the potential energy surface. Solvent effects were taken into account by the polarizable continuum model (PCM), using parameters for water [44]. The hydrogen atom transfer (HAT) mechanism is governed by the homolytic bond dissociation enthalpy (BDE) [24], while the single electron transfer (SET) mechanism is governed by the ionization potential (IP) [37].

BDE values were calculated for the homolytic breakage of each O-H and N-H bond present in the compounds, as follows:

(2) $$BDE=H\left(RX|•\right)+H\left(H|•\right)-H$$

where *H* (RXH), *H* (RX•), and *H* (H•) are the enthalpies of the neutral molecules, their radicals, and the H-atom, respectively [37].

IP values were calculated using the adiabatic approach as follows:

(3) $${IP}^{adiab}=H_{N-1}\left(g_{N-1}\right)-H_{N}\left(g_{N}\right)$$

where *H_N_*(*g_N_*) and *H_N_*_−1_(*g_N_*_−1_) are the enthalpies of the neutral and cationic forms of the compound, optimized at the corresponding form.

### 3.5. Phenolic Profile Analysis

#### 3.5.1. Preparation of Standard Solutions

Stock standard solutions of all the phenolics (1 mg/mL) were prepared by separately dissolving a precise amount of each standard in HPLC-grade methanol with a sonication aid (for a few minutes). Care was taken to ensure that the water bath temperature during sonication did not increase above 30 °C. The stock solutions were stored in the dark at −20 °C. Working standard solutions that contained all the phenolics were prepared, diluting the standard stock solutions with the initial mobile phase. The standard solutions were sonicated/vortexed after preparation and before use (injection in the HPLC) to ensure the maximal solubilization of each compound in the mixture.

#### 3.5.2. High-Performance Liquid Chromatography

Liquid chromatography procedures were carried out as described by Soto et al. [45], with some modifications. Analyses were performed on a Knauer Azura analytical UHPLC system (Knauer, Berlin, Germany), consisting of an Azura P 6.1 L pump, 3950 autosamplers, and an Azura DAD 2.1 L diode array detector with high-sensitivity Knauer. A reversed-phase column (Luna^®^, 100 mm × 4.6 mm, 3.0 µm, Phenomenex, Torrance, CA, USA) was used, and the temperature of the column was kept at 25 °C. The mobile phase consisted of two solvents: (A) 1% formic acid in water and (B) acetonitrile. The following gradient program was run: at 0 min, the A:B ratio was 95:5 *v*:*v*; at 5 min, 70:30; at 10 min, 30:70; and at 22 min, 95:5. The system was allowed to run for another 10 min to equilibrate the column before each injection (95:5). The flow rate of the mobile phase was 0.7 mL/min. The detection wavelengths were 260, 270, 280, 320, 330, and 360 nm, and DAD was recorded from 200 to 500 nm for peak characterization. Calibration standards were prepared by diluting a concentrated mixture solution with an initial mobile phase in the concentration range of 0.5–20 µg/mL for the rest of the acid phenolics. The most likely identification of phenolics was achieved by comparing the retention times and HPLC spectra of each peak in the sample with those of the respective phenolic compound standards.

### 3.6. Antibacterial Activity

#### 3.6.1. Strain and Growth Conditions

The *S. nutans* ethanolic extract was used to determine its antibacterial activity against human pathogenic bacteria *Bacillus subtilis* (ATCC 6051), *Escherichia coli* (ATCC 23716), *Salmonella enterica* (ATCC 13311), *Pseudomonas aeruginosa* (ATCC 19429), and *Staphylococcus aureus* (ATCC 29737) and the phytopathogenic bacteria *Agrobacterium tumefaciens* (ATCC 19358), *Erwinia rhapontici* (MK883065), *Pantoea agglomerans* (MK883087), *Pseudomonas syringae* (MF547632), and *Xanthomonas campestris* (MH885473). Bacteria were cultivated in nutrient broth containing 5.0 g/L peptone and 3.0 g/L meat extract and incubated at 25 °C (phytopathogens) or 35 °C (human pathogens) for 18 h at 150 rpm using an incubator with orbital shaking, LOM-80 (MRC Lab, London, UK).

#### 3.6.2. Minimum Inhibitory Concentration (MIC)

The MIC of the *S. nutans* extract was determined by the method described by Simirgiotis et al. [46] in order to determine the minimum concentration necessary to inhibit bacteria. Working concentrations ranged from 0 to 10 mg/mL of the ethanolic extract. For this purpose, a stock solution of 20 mg/mL of extract was prepared. Further dilutions were prepared in nutrient broth prior to inoculation with each bacterium. Dilutions were prepared to a final working volume of 200 µL in 96-well plates and inoculated with the different bacteria to be tested at 25 or 35 °C, as appropriate. A growth control consisting of nutrient broth without extract and inoculated with each bacterium was used. A negative control of the growth sterility of the *S. nutans* dilution was carried out in nutrient broth with 0–10 mg/mL of *S. nutans* extract without bacteria. An additional assay using 0–90% (*v*/*v*) ethanol was performed to estimate the ethanol MIC for each bacterium. Concentrations higher than 20% ethanol were inhibitory for all bacteria. After 24 h of incubation, the MIC was determined from the lowest extract concentration where no bacterial growth was observed.

#### 3.6.3. Minimum Bactericidal Concentration (MBC)

The MBC of the ethanolic extract of *S. nutans* was determined from the last three wells where no bacterial growth was observed in the MIC assay, as described by Simirgiotis et al. [46]. For this purpose, 100 µL from these wells was inoculated in nutrient broth plates supplemented with 1.5% agar. The plates were incubated for 24 h at the corresponding temperature, after which the MBC of the *S. nutans* extract was determined from nutrient agar plates where no growth was observed. A culture with no bacterial inhibitory growth in the MIC test was used as a growth control.

### 3.7. Statistical Analysis

The statistical analysis was carried out using the OriginPro 9.1 software packages (OriginLab Corporation, Northampton, MA, USA). The Tukey and Fisher comparison tests determined significant differences between means (*p* values < 0.05 were considered significant).

## 4. Conclusions

The micropropagation and ex vitro adaptation techniques were successfully employed to conserve *S. nutans*, an endemic Atacama Desert Andean Region species. Micropropagation through stem cuttings allowed controlled growth independent of seasonal and climatic variations. The regenerated plants exhibited robust growth and high survival rates during acclimation and transplantation. UHPLC-DAD analysis revealed no significant changes in the phenolic profile, with prenylated acetophenone being the most abundant metabolite. For the first time, 4-hydroxybenzoic acid, vanillic acid, and cinnamic acid were identified in the ethanolic extract from ex vitro cultures of *S. nutans*. In vitro cultivation reduced the phenol and flavonoid content and antioxidant and antibacterial potential. However, these characteristics were significantly restored after adaptation to ex vitro conditions. Independently of the applied culture condition, the ethanolic extracts of *S. nutans* presented high activity against both Gram-positive and Gram-negative bacteria, demonstrating their antimicrobial capacity. This successful initiation of in vitro and ex vitro cultures provides a biotechnological approach for the conservation of *S. nutans* and ensures a reliable and consistent source of AC as a potential raw material for pharmacological applications.

## Figures and Tables

**Table 1:** Phenolic compounds identified in the extracts of *S. nutans*.

Peak	Phenolic Compound *	rt	Wild	Ex Vitro	In Vitro
			(mg/100 g of DW)		
1	4-Hydroxybenzoic acid	4.38	280.03 ± 22.43 ^a^	93.12 ± 15.31 ^b^	47.89 ± 9.14 ^c^
2	Vanillic acid	4.82	116.09 ± 12.52 ^a^	146.39 ± 14.43 ^b^	64.62 ± 11.25 ^c^
3	Ferulic acid	6.75	82.10 ± 12.01 ^a^	31.03 ± 8.98 ^b^	14.21 ± 5.10 ^c^
4	4-Hydroxy-3-(3-methyl-2-butenyl) acetophenone	12.70	4356.22 ± 84.99 ^a^	4339.30 ± 93.12 ^a^	4202.73 ± 99.35 ^a^
5	Chlorogenic acid	13.72	24.21 ± 6.64 ^a^	31.00 ± 6.19 ^a^	20.52 ± 4.87 ^a^
6	Cinnamic acid	13.98	44.56 ± 8.98 ^a^	82.54 ± 18.13 ^b^	18.77 ± 2.66 ^c^

* Identified by spiking experiments with authentic standards or isolated compounds. Values that have different superscript letters differ significantly (*p* < 0.05).

**Table 2:** Hydroxyl bond dissociation enthalpy (BDE) and ionization potential (IP) for AC, calculated at M06-2x/6-311+G(d,p) level.

Compound	BDE (kcal/mol)	IP (kcal/mol)
**4**	86.14	186.34

**Table 3:** Antibacterial activity of *Senecio nutans* ethanolic extract.

Bacterium		MIC ^1^ (mg/mL)						MBC ^2^ (mg/mL)		
	IS	EV	IV	4	Kan ^3^	IS	EV	IV	4	Kan ^3^
*Escherichia coli* (ATCC 23716)	0.625	1.25	1.25	1.25	5.00	5.00	5.00	5.00	5.00	10.00
*Pseudomonas aeruginosa* (ATCC 19429)	1.25	1.25	1.25	0.625	5.00	5.00	5.00	5.00	2.50	10.00
*Salmonella enterica* (ATCC 13311)	0.313	0.313	1.25	0.16	2.50	2.50	2.50	2.50	0.625	5.00
*Bacillus subtilis* (ATCC 6051)	0.625	0.625	0.625	0.16	1.25	N.D.	N.D.	N.D.	0.625	20.00
*Staphylococcus aureus* (ATCC 29737)	0.313	0.625	1.25	0.16	2.50	2.50	2.50	2.50	0.313	10.00
*Erwinia rhapontici* (MK883065)	0.625	0.625	1.25	0.625	1.25	5.00	5.00	5.00	2.50	2.50
*Pseudomonas syringae* (MF547632)	0.625	0.625	1.25	0.625	1.25	2.50	2.50	2.50	2.50	2.50
*Pantoea agglomerans* (MK883087)	0.625	0.625	0.625	0.625	2.50	5.00	5.00	5.00	2.50	5.00
*Agrobacterium tumefaciens* (ATCC 19358)	1.25	1.25	1.25	0.08	1.25	2.50	2.50	2.50	0.313	5.00

^1^ Minimum inhibitory concentration. ^2^ Minimum bactericidal concentration. N.D.: inhibition not detected. ATCC: American Type Culture Collection (USA). MK883065, MF547632, MK883087, and MH885473 are the accession numbers in GenBank of the respective bacteria. ^3^ The kanamycin concentration is expressed as µg/mL.

## Data Availability

Data are contained within the article.

## Supplementary Materials

The following supporting information can be downloaded at https://www.mdpi.com/article/10.3390/plants13060755/s1. Table S1. Morphogenic responses of *Senecio nutans* explants cultured in MS medium with 2-iP in the explant establishment stage. Table S2. Morphogenic responses of *Senecio nutans* shoots, obtained in vitro and cultivated in MS medium with 2-iP in the proliferation stage. Table S3. Morphogenic responses of *Senecio nutans* shoots obtained in vitro and cultivated in MS medium with IBA at the rooting stage. Optimized geometries at DFT M06-2x/6-311+G(d,p) level of AC.
