# Supplementary material for: Effect of In Vitro Micropropagation on the Chemical, Antioxidant, and Biological Characteristics of Senecio nutans Sch. Bip., an Endemic Plant of the Atacama Desert Andean Region
Source: Plants (Basel). 2024 Mar 7;13(6):755. doi: 10.3390/plants13060755 (PMC10976176; doi:10.3390/plants13060755)
Supplement: Supplementary file 1 [file plants-13-00755-s001.zip › plants-2857187-supplementary.pdf]

Supporting Information

Effect of In Vitro Micropropagation on the Chemical, Antioxidant, and Biological Characteristics of Senecio nutans Sch. Bip., an Endemic Plant of the Atacama Desert Andean Region

Claudio Parra [1,*] , Patricio Munoz-Torres [2], Hugo Escobar [2], Mario J. Simirgiotis [3], Gabriela Contreras-Contreras [4], Álvaro Ruiz-Fernández [5] , Cristian Maulen [1] , Maximiliano Martínez-Cifuentes [1,*] and María Salomé Mariotti-Celis [4,*]

[1] Departamento de Química Orgánica, Facultad de Ciencias Químicas, Universidad de Concepción, Concepción 4070371, Chile; cmaulen2018@udec.cl
[2] Facultad de Ciencias Agronómicas, Universidad de Tarapacá, Arica 1000000, Chile; pmunozt@academicos.uta.cl (P.M.-T.); hescobar@academicos.uta.cl (H.E.)
[3] Facultad de Ciencias, Instituto de Farmacia, Universidad Austral de Chile, Valdivia 5110566, Chile; mario.simirgiotis@uach.cl
[4] Escuela de Nutrición y Dietética, Facultad de Medicina, Universidad Finis Terrae, Santiago 7501015, Chile; gcontreras@uft.cl
[5] Computational Biology Lab, Fundación Ciencia & Vida, Santiago 8580702, Chile; aruiz@dlab.cl
* Correspondence: cparra@udec.cl (C.P.); maxmartinez@udec.cl (M.M.-C.); mmariotti@uft.cl (M.S.M.-C.)

Table S1. Morphogenic responses of *Senecio nutans* explants cultured in MS medium with 2-iP in the explant establishment stage

2-iP (μM)	Survival (%)	Sprouting (%)	No. shoots/explant	Rooting (%)
-	40.1 ± 5.6 [d]	50.8 ± 4.8 [c]	1.8 ± 0.3 [b]	57.2 ± 3.3 [b]
2.5	60.3 ± 6.5 [bc]	67.5 ± 4.7 [b]	2.4 ± 0.3 [a]	57.2 ± 3.3 [b]
3,5	63.1 ± 5,6 [bc]	69.5 ± 4.1 [b]	2.4 ± 0.3 [a]	58.6 ± 2.8 [b]
5.0	77.9 ± 13.9 [a]	85.4 ± 9.2 [a]	2.5 ± 0.4 [a]	64.6 ± 4.9 [a]
7.0	71.9 ± 12.0 [a]	85.4 ± 9.2 [a]	2.5 ± 0.4 [a]	61.7 ± 2.0 [ab]
10.0	55.1 ± 8.5 [c]	84.0 ± 12.1 [a]	2.9 ± 0.5 [a]	58.0 ± 4.5 [b]

2-iP: 2-Isopentyl adenine. Values are the mean of three replicates ± SD. Means with different letters differ significantly by ANOVA followed by Fisher's test ($p < 0.05$).

Table S2. Morphogenic responses of *Senecio nutans* shoots, obtained *in vitro* and cultivated in MS medium with 2-iP in the proliferation stage.

2-iP (μM)	Survival (%)	Sprouting (%)	No. shoots/explant	Rooting (%)
-	45.0 ± 7.9 [b]	47.4 ± 4.8 [c]	2.6 ± 0.5 [b]	29.6 ± 6.5 [c]
2.5	77.9 ± 13.9 [a]	68.7 ± 3.3 [b]	4.6 ± 0.7 [a]	59.4 ± 3.8 [b]
3,5	84.0 ± 12.1 [a]	74.8 ± 10.5 [b]	4.5 ± 0.7 [a]	62.3 ± 7.2 [b]
5.0	84.0 ± 12.0 [a]	90.0 ± 0.0 [a]	4.6 ± 0.5 [a]	74.1 ± 11.2 [a]
7.0	95.8 ±12.1 [a]	85.4 ± 9.2 [a]	4.6 ± 0.5 [a]	60.3 ± 4.3 [b]
10.0	84.0 ± 12.0 [a]	90.0 ± 0.0 [a]	4.5 ± 0.8 [a]	53.4 ± 1.8 [b]

2-iP: 2-Isopentyl adenine. Values are the mean of three replicates ± SD. Means with different letters differ significantly by ANOVA followed by Fisher's test ($p < 0.05$).

Table S3. Morphogenic responses of *Senecio nutans* shoots obtained *in vitro* and cultivated in MS medium with IBA at the rooting stage.

IBA (μM)	Survival (%)	Sprouting (%)	No. shoots/explant	Rooting (%)
-	100	100	1.5 ± 0.2 [b]	40.1 ± 5.6 [a]
2.5	100	100	1.5 ± 0.1 [b]	27.8 ± 6.5 [b]
3,5	100	100	1.6 ± 0.1 [b]	26.8 ± 5.6 [b]
5.0	100	100	1.5 ± 0.1 [b]	27.1 ± 5.3 [b]
7.0	100	100	1.6 ± 0.1 [b]	40.1 ± 5.6 [a]
10.0	100	100	1.9 ± 0.2 [a]	47.3 ± 4.7 [a]

IBA: Indole-3-butyric acid. Values are the mean of three replicates ± SD. Means with different letters differ significantly by ANOVA followed by Fisher's test ($p < 0.05$).

Optimized geometries at DFT M06-2x/6-311+G(d,p) level

4-hydroxy-3-(3-methyl-2-butenyl) acetophenone

Neutral
0 1

C	1.43739400	2.20982700	-0.43901900
C	2.35666400	1.17871200	-0.52668000
C	2.03652400	-0.09179100	-0.04745300
C	0.77884600	-0.30067200	0.52447500
C	-0.16011400	0.71768500	0.62841300
C	0.18574800	1.97843300	0.13071000
H	1.68364700	3.19919600	-0.81243600
H	3.33364600	1.33702500	-0.96721100
H	0.50831400	-1.28117500	0.90303600
C	3.05699100	-1.17762100	-0.16936000
C	-1.53364400	0.47998500	1.21410400
H	-1.80933800	1.33867000	1.83229600
H	-1.49430200	-0.39128200	1.86772200

C	-2.56593000	0.30998200	0.12764700
H	-2.80271000	1.22237900	-0.41489800
C	-3.17317000	-0.81865200	-0.24507900
C	-2.94487300	-2.17205900	0.37302500
H	-2.56910100	-2.86862700	-0.38349000
H	-2.23790200	-2.15982600	1.20056800
H	-3.89015500	-2.58494800	0.73915200
C	-4.17084300	-0.82118300	-1.37343900
H	-5.14470200	-1.18096500	-1.02623800
H	-4.30184100	0.17460000	-1.79790100
H	-3.84743500	-1.49783900	-2.17093400
O	-0.75367000	2.95815500	0.23175400
H	-0.41075000	3.77952200	-0.13109700
O	4.13809900	-0.95906600	-0.66738400
C	2.70531100	-2.55788200	0.34247600
H	2.49143700	-2.52576400	1.41353200
H	1.81575500	-2.94029800	-0.16360800
H	3.54658800	-3.22196200	0.15763000

Radical Cation

1 2

C	-0.83583000	2.29965600	0.60361100
C	-1.85838500	1.38007100	0.66701000
C	-1.75973200	0.11968800	0.02536500
C	-0.60943700	-0.20406500	-0.65380000
C	0.46154500	0.71450100	-0.74541000
C	0.33294500	1.97624800	-0.08978900
H	-0.92818400	3.26667300	1.08619300
H	-2.77226900	1.60244000	1.20671100
H	-0.52465000	-1.15107400	-1.17422100
C	-2.94961600	-0.80923000	0.12361400
C	1.72331500	0.40209000	-1.49976300
H	2.16229700	1.27546600	-1.97447100
H	1.59389200	-0.41027800	-2.20860600
C	2.41348700	0.03484600	-0.22782800
H	2.84514600	0.86642600	0.32079000
C	2.49643300	-1.22341800	0.33715100
C	1.86952000	-2.44219000	-0.24642300
H	0.97917200	-2.69852600	0.34219100
H	1.57607400	-2.33180200	-1.28835700
H	2.55087600	-3.29148700	-0.15712100
C	3.26237800	-1.41288400	1.60057700
H	4.13562100	-2.04559500	1.40556700
H	3.59908200	-0.47367000	2.03549000
H	2.65097600	-1.95584000	2.32937500
O	1.37113100	2.80551600	-0.20444100
H	1.19953500	3.65846900	0.21372800
O	-3.87979200	-0.48377400	0.81409500
C	-2.92181700	-2.10372200	-0.64590000
H	-2.80424000	-1.91326200	-1.71566700

H	-2.08696700	-2.72932700	-0.31849400
H	-3.85590900	-2.63209800	-0.47082800

Neutral Radical

0 2

C	-1.25855500	2.25173600	0.55416300
C	-2.20476400	1.27668000	0.62675800
C	-1.96975700	0.00406300	0.04249800
C	-0.75742200	-0.26063800	-0.61680200
C	0.23067000	0.69367600	-0.71430400
C	0.00975700	2.01766100	-0.12018100
H	-1.40433400	3.23453100	0.98670400
H	-3.15350700	1.43718000	1.12420400
H	-0.58806700	-1.23604900	-1.06069700
C	-3.04688600	-1.02988000	0.16112500
C	1.56106500	0.43034800	-1.35665900
H	1.82107700	1.28137900	-1.99017100
H	1.49767200	-0.45750800	-1.98654200
C	2.62277400	0.28414400	-0.28694800
H	3.02725600	1.22334800	0.07977600
C	3.04796500	-0.85717400	0.25858100
C	2.53884600	-2.22546000	-0.10560300
H	2.03863200	-2.67818600	0.75696800
H	1.83626900	-2.21877900	-0.93752700
H	3.37274800	-2.88352500	-0.36785800
C	4.09814700	-0.85254800	1.33695300
H	4.97372100	-1.43075700	1.02524200
H	4.42033200	0.16002900	1.58022300
H	3.71382200	-1.32245800	2.24796300
O	0.87431800	2.89892800	-0.20099600
O	-4.07964400	-0.76383000	0.73265000
C	-2.81296400	-2.39758200	-0.43839500
H	-2.65783800	-2.32266500	-1.51741300
H	-1.92398300	-2.86132700	-0.00444500
H	-3.68444300	-3.01614200	-0.23677900
